# MicroRNA-107: a novel promoter of tumor progression that targets the CPEB3/EGFR axis in human hepatocellular carcinoma

**DOI:** 10.18632/oncotarget.5689

**Published:** 2015-10-17

**Authors:** Chen-Dan Zou, Wei-Ming Zhao, Xiao-Na Wang, Qiang Li, Hui Huang, Wan-Peng Cheng, Jian-Feng Jin, He Zhang, Ming-Juan Wu, Sheng Tai, Chao-Xia Zou, Xu Gao

**Affiliations:** ^1^ Department of Biochemistry and Molecular Biology, Harbin Medical University, Harbin, China; ^2^ Department of General Surgery, the Second Affiliated Hospital of Harbin Medical University, Harbin, China; ^3^ Academy of Traditional Chinese Medicines, Harbin, China; ^4^ Heilongjiang Academy of Medical Science, Harbin, China

**Keywords:** hepatocellular carcinoma, microRNA, microRNA-107, CPEB3, EGFR

## Abstract

MicroRNAs (miRNAs) are dysregulated in many types of malignancies, including human hepatocellular carcinoma (HCC). MiR-107 has been implicated in several types of cancer regulation; however, relatively little is known about miR-107 in human HCC. In the present study, we showed that the overexpression of miR-107 accelerates the tumor progression of HCC *in vitro* and *in vivo* through its new target gene, CPEB3. Furthermore, our results demonstrated that CPEB3 is a newly discovered tumor suppressor that acts via the EGFR pathway. Therefore, our study demonstrates that the newly discovered miR-107/CPEB3/EGFR axis plays an important role in HCC progression and might represent a new potential therapeutic target for HCC treatment.

## INTRODUCTION

Hepatocellular carcinoma (HCC) is one of the most prevalent malignancies and a leading cause of cancer mortality worldwide [1–4]. Although several studies have identified a few genes that are involved in the progression of this malignancy, the pathogenesis of HCC remains to be elucidated [5, 6].

MiRNAs are a class of 17–24-bases non-coding RNA molecules that have become widely characterized as a critical regulator in the development and progression of cancer [7–10]. Recent studies have shown that specific miRNAs could have important influence on hepatic carcinogenesis and directly contribute to cell proliferation and metastasis in HCC [11–13]. Although miR-107 has been identified as an upregulated gene in HCC tissue compared with non-tumor tissue based on microarray analysis, relatively little is known about the role of miR-107 in human HCC [17]. Here, we methodically investigated the biological functions and underlying molecular mechanisms of miR-107 in human HCC progression. We demonstrated that the overexpression of miR-107 promotes human HCC cell proliferation, migration and invasion.

Cytoplasmic polyadenylation element binding protein 3 (CPEB3) is a member of the CPEBs family, which can regulate translation by modulating cytoplasmic polyadenylation. A microarray analysis demonstrated that the CPEB3 level is decreased in HCC tissues versus healthy controls [18]. Although CPEB3 has been mentioned in several studies, no direct functional study on its role in cancer has been performed. In the present study, we showed that miR-107 promotes the progression of HCC by targeting the CPEB3/EGFR axis and that this newly discovered mechanism might provide a new potential therapeutic target for HCC treatment.

## RESULTS

### miR-107 promotes human HCC cell proliferation *in vitro* and *in vivo*

**Figure 1:**
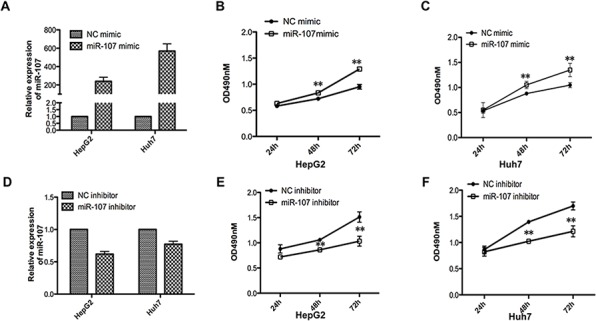
miR-107 promotes cell growth ability *in vitro* **A.** Relative miR-107 expression levels of the two cell lines (HepG2 and Huh7) were detected with quantitative real-time PCR (qRT-PCR) after transfecting with the miR-107 mimic or the negative control (NC mimic). **B–C.** MTT assays were performed with the HepG2 (B) and Huh7 (C) cells transfected with the NC mimic or the miR-107 mimic **D–F.** The same experiments as (A-C) were performed in cells transfected with miR-107 inhibitor and negative control (inhibitor NC). Points are the average of three independent experiments; bars represent the standard error. Data represent the average of three independent experiments (*P < 0.05; **P < 0.01; ***P < 0.001).

**Figure 2:**
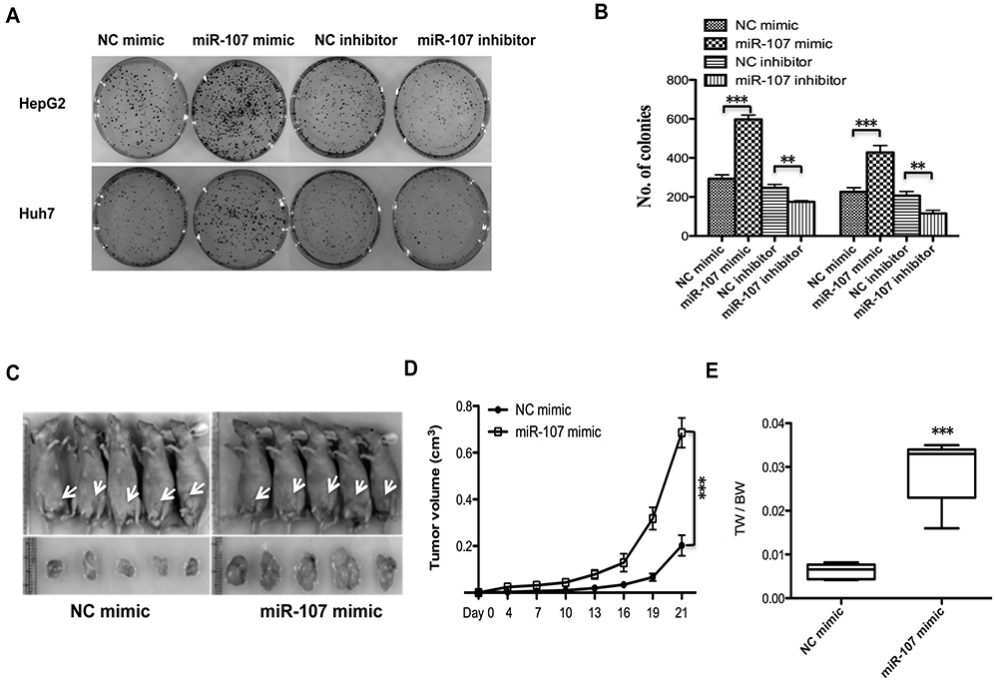
miR-107 promotes tumor proliferation *in vitro* and in a xenograft model **A–B.** Colony formation assays. Cells were transfected with NC mimic/miR-107 mimic and NC inhibitor/miR-107 inhibitor. **C.** Tumors formed in nude mice. A total 5 × 10^6^ cells were subcutaneously injected into the nude mice (*n* = 5). The mice were then sacrificed 21 days after the injection. Tumors were harvested, and images of representative tumors were obtained. **D.** The tumor volumes were determined, and miR-107 overexpression resulted in an increased growth rate. **E.** Tumors and the bodies were weighted and the data are expressed as tumor weight/body weight (TW/BW) (*P < 0.05; **P < 0.01; ***P < 0.001).

To investigate the biological function of miR-107 in HCC progression, we performed MTT assays with two human HCC cell lines (HepG2 and Huh7) that were transfected with a miR-107 mimic and its negative control (NC mimic)(Figure 1A). The results demonstrated that the overexpression of miR-107 significantly promoted cell proliferation in both cell lines (Figure 1B–1C). Moreover, the growth ability of cells decreased when endogenous miR-107 was silenced with a miRNA inhibitor (Figure 1D–1F). Additionally, colony formation assays were performed. We found that the growth ability was increased in miR-107-overexpressing cells compared with the NC group (Figure 2A–2B). These results indicate that miR-107 significantly accelerates human HCC cell proliferation *in vitro*.

To explore the role of miR-107 in tumor proliferation *in vivo*, the xenograft model of human HCC cells in nude mice was used. HepG2 cells transfected with NC mimic/miR-107 mimic were injected subcutaneously into the flank of each nude mouse. The tumor size was measured every day, and the growth curve against the average tumor size was plotted. After 3 weeks, all of the mice were sacrificed, and their bodies and the xenografts were weighed. As expected, there was a significant increase in tumor size and weight of the miR-107-overexpressing groups compared with the NC group (Figure 2C–2E). Taken together, these observations suggest that miR-107 is a positive proliferative regulator in HCC.

### Overexpression of miR-107 exacerbates human HCC cell migration and invasion

**Figure 3:**
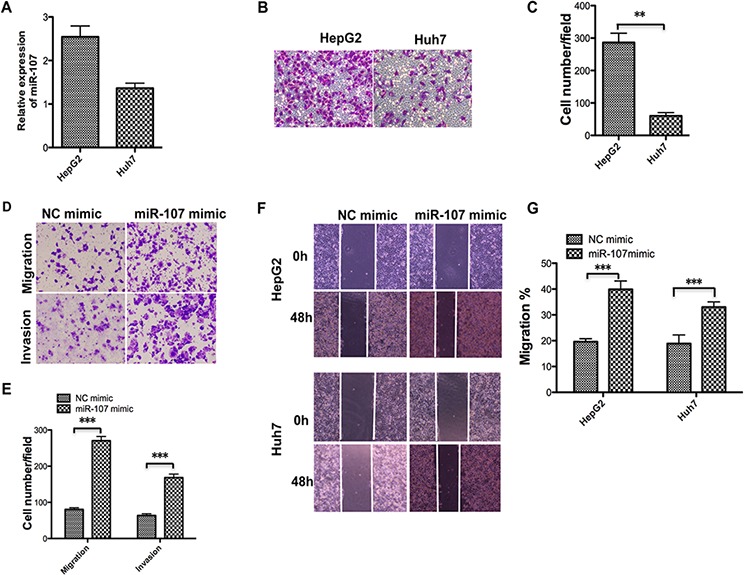
Overexpression of miR-107 promotes the migration and invasion of human HCC cells **A–C.** The miR-107 level was detected using qRT-PCR (A) in the two cell lines and the transwell assay without Matrigel was performed. Representative photos (B) and quantitative analysis (C) are shown. **D–E.** Transwell assays with and without Matrigel were performed with the Huh7 cells transfected with the NC mimic and the miR-107 mimic. Representative photos (D) and quantitative analysis (E) are shown. **F–G.** Wound-healing assays were performed to evaluate the effect of miR-107 on the migratory ability of the two cell lines. The artificial gap was made through the central axis when the cells reached a density of 80%. Photos of the cells were taken at 0 and 48 h (F) The relative migration length was from three randomly selected locations (G) (*P < 0.05; **P < 0.01; ***P < 0.001).

To better understand miR-107 function in human HCC cell metastasis, we tested the miR-107 level and migratory ability of the two cell lines (Figure 3A–3C). We then selected Huh7 to perform the subsequent transwell assays with and without Matrigel assays to obtain more observable results. The transwell assays without Matrigel demonstrated that overexpression of miR-107 by transfecting its mimic significantly promotes the migration of Huh7 compared with the NC group. The transwell assays with Matrigel showed that the invasive capacity was dramatically enhanced in these cells compared with the NC group (Figure 3D–3E). Furthermore, a wound-healing assay was performed to confirm the effect of miR-107 on metastasis. The results showed that overexpression of miR-107 increased the migration rate of both cell lines (Figure 3F–3G). These findings indicate that miR-107 promotes migration and invasion of HCC cells.

### miR-107 downregulates CPEB3 expression by directly targeting the CPEB3 3′-UTR

**Figure 4:**
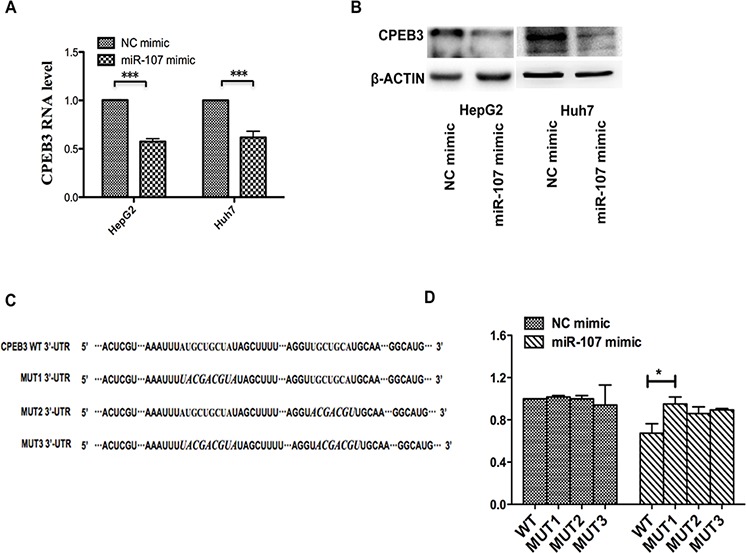
miR-107 directly targets the CPEB3 3′-UTR **A–B.** qRT-PCR and western blotting were applied to detect the mRNA and protein expression levels of CPEB3 in the HepG2 and Huh7 cells transfected with the NC mimic and miR-107 mimic. **C.** The prediction targeting site of CPEB3 3′-UTR combined with miR-107 were shown (MUT1, the first binding site is mutant; MUT2, the second binding site is mutant and MUT3, both binding sites are mutant). **D.** HEK-293T cells were cotransfected with wild-type 3′-UTR (WT-UTR) or mutant-type 3′-UTR (MUT1, MUT2 and MUT3) reporters and the NC mimic or the miR-107 mimic. In the experiments shown in the panels, luciferase/Renilla activity was measured (*P < 0.05; **P < 0.01; ***P < 0.001).

To understand how miR-107 mediates human HCC cell growth and metastasis, bioinformatics strategies were used to search for the potential targets of miR-107. All four bioinformatics algorithms, miRanda, TargetScan, miRBase, and PicTar, indicated that CPEB3 was a target of miR-107. In addition, the high expression of miR-107 dramatically suppressed the endogenous mRNA and protein levels of CPEB3 in human HCC cell lines (Figure 4A–4B). To determine whether CPEB3 is directly targeted by miR-107 at its 3′-UTR, the luciferase reporter plasmid containing 3′-UTR fragments of CPEB3 was co-transfected with miR-107 mimics and NC mimics. There are two predicted miR-107 binding sites in the 3′-UTR of CPEB3, so we built different 3′-UTR fragments, including wild-type (WT-UTR) and mutant-type 3′-UTR (MUT1, MUT2 and MUT3) to clarify the functional site (Figure 4C). As shown in Figure 4D, the relative luciferase activity was remarkably reduced by miR-107 when the wild-type 3′-UTR of CPEB3 was present. This reduction was sequence-specific because the relative luciferase activity in mutant-type UTR did not drop as sharply as that in wild-type UTRs. However, we cannot determine the specific site for this binding based on this result. These data indicate that miR-107 directly targets the CPEB3 3′UTR, thereby reducing CPEB3 expression.

### Downregulated CPEB3 promotes human HCC cell proliferation and metastasis

**Figure 5:**
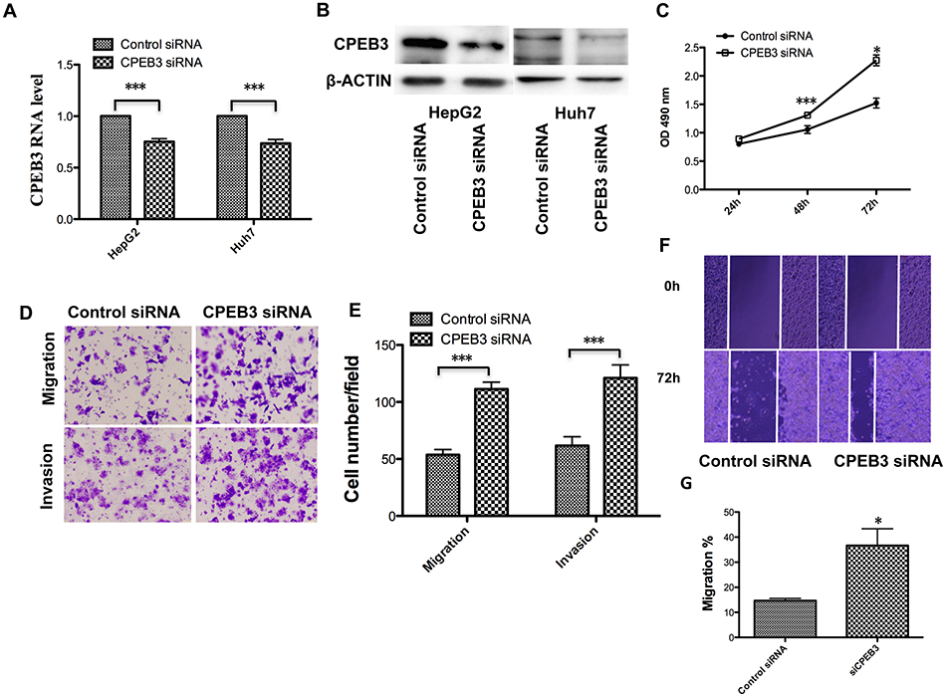
CPEB3 inhibition accelerates the proliferation and metastasis of human HCC cells **A.** The Huh7 and HepG2 cells were transfected with negative control (siNC) and CPEB3 siRNA (siCPEB3). The mRNA level and protein level of CPEB3 after interference were detected using qRT-PCR and western blotting. **C.** Huh7 cells were used in the MTT assay after transfection of siNC and siCPEB3. **D–E.** Downregulated CPEB3 promoted the migratory and invasive ability of the Huh7 cells. The transwell assays with and without Matrigel were performed in Huh7 cells transfected siNC and siCPEB3. Representative photos (D) and quantitative analysis (E) are shown. A wound-healing assay was performed using the same cells as is (D), and the photos were taken at 0 h and 72 h **F–G.** Relative migration length was from three randomly selected locations (*P < 0.05; **P < 0.01; ***P < 0.001).

**Figure 6:**
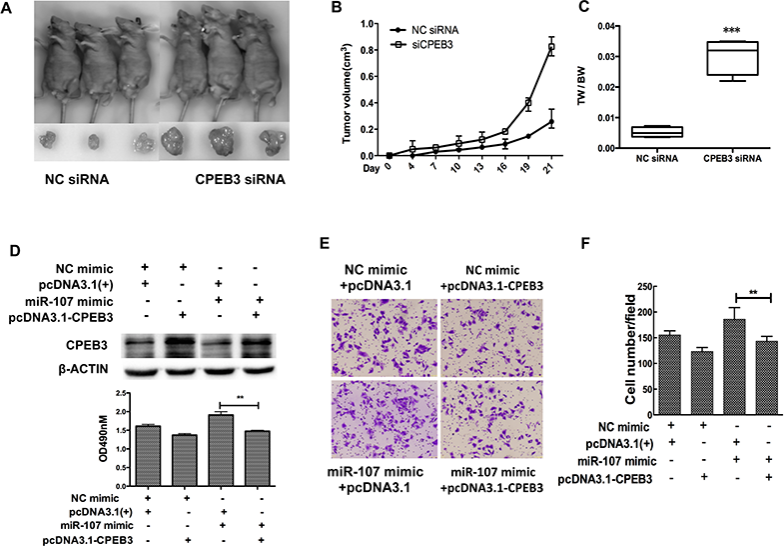
CPEB3 is involved in miR-107-induced growth and migration in HCC cells **A.** Tumors formed in nude mice. The hepG2 cells transfected with CPEB3 siRNA and negative control were subcutaneously injected into nude mice. **B.** The volumes of tumors were determined and CPEB3 interfering resulted in the promotion of the growth rate. **C.** Tumors and the bodies were weighted and showed as tumor weight/body weight (TW/BW). **D.** Huh7 cells were co-transfected with the miR-107 mimic (or NC mimic) and the CPEB3 expression vector (or pcDNA 3.1(+)). The expression of CPEB3 was detected by western blot. **E–G.** The cells transfected as in (D) were used to perform the MTT and transwell assays (*P < 0.05; **P < 0.01; ***P < 0.001).

Previous reports indicated that CPEB3, as a member of the CPEB family, plays an important role in the nervous system, especially the biological functions associated with memory [19, 20]. However, few studies have addressed the biological function of CPEB3 in cancer. To gain insight into the potential role of CPEB3 in HCC, Huh7 cells were transfected with CPEB3 siRNA (Figure 5A–5B) to perform the MTT, transwell and the wound healing assays. Remarkably, silencing of CPEB3 using siRNA strongly promoted the proliferation of the cells (Figure 5C). The ability of migration and invasion of the cells was enhanced (Figure 5D–5G) as a result of the overexpression of miR-107. Furthermore, the xenograft model of human HCC cells in nude mice was applied. HepG2 cells transfected with Control siRNA or CPEB3 siRNA were injected subcutaneously into the flank of each nude mouse. As expected, there was a significant increase in tumor size and weight of CPEB3 siRNA groups compared with the NC group (Figure 6A–6C).

We have previously demonstrated miR-107 downregulated CPEB3 at the mRNA and protein levels, so we hypothesized that *in vitro* phenotypes associated with miR-107 could be reversed via the restoration of CPEB3 levels. To test this hypothesis, we constructed a CPEB3 expression vector, then co-transfected the CPEB3 expression vector (or empty vector) with the miR-107 mimic (or NC mimic) into the Huh7 cells. The expression of CPEB3 was confirmed by western blot analysis (Figure 6D). Importantly, the MTT and transwell assays indicated that the restoration of CPEB3 significantly decreased miR-107-induced proliferation (Figure 6D) and migration (Figure 6E–6F).

As a result, these findings suggest that miR-107 functionally targets CPEB3 and promotes tumor effects partially through CPEB3.

### EGFR is involved in the miR-107 pathogenesis of HCC through CPEB3

**Figure 7:**
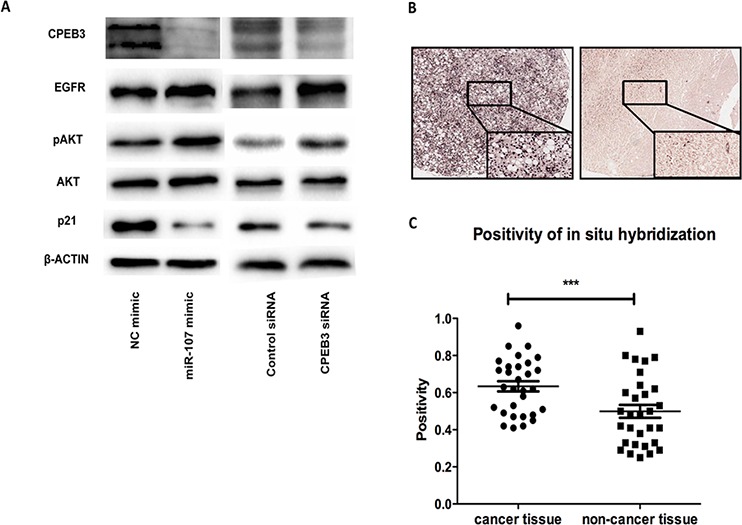
The involved pathway and clinical analysis **A.** miR-107 mediates CPEB3 inhibitory functions through the EGFR pathway. The protein levels of CPEB3, EGFR, AKT, pAKT, p21 and β-actin were tested by western blotting in the Huh7 cells. **B.** Representative results of the *in situ* hybridization of HCC specimens and corresponding adjacent non-cancerous liver tissues for miR-107. **C.** miR-107 was detected in both HCC tissues and normal tissues. The miR-107 positivity analysis is shown.

To understand the underlying mechanisms of the miR-107 oncogenic effects in HCC, we performed western blotting in the HepG2 and Huh7 cells to explore the correlative genes in HCC. Given that the epidermal growth factor receptor (EGFR) has been demonstrated to be the regulative pivot of CPEB3 in the nervous system in previous studies [19], we tested the expression of EGFR in human HCC cells. Interestingly, we found that the CPEB3 expression decreased by miR-107 was accompanied by the upregulation of EGFR and pAKT. Then, the reduction of p21 was successively confirmed to be involved in this pathogenesis (Figure 7A). We also tested the PTEN level, as a putative HCC suppressor [21], but found no significant correlation with miR-107 or CPEB3 (data not shown). Therefore, these observations suggest that miR-107 is a newly discovered HCC promoter that is associated with EGFR signaling pathway partially through its target, CPEB3.

### High expression of miR-107 was observed in cancer tissue compared with non-cancer tissue in clinical samples

To test whether miR-107 plays the same role, a HCC promoter, in clinical samples, we evaluated the expression of miR-107 using *in situ* hybridization (ISH) on tissue microarrays containing 30 HCC tissues and corresponding non-cancerous liver tissues. The expression possibility of miR-107 was significantly higher in the HCC tissues than in the corresponding non-cancerous liver tissue (Figure 7B–7C). These results suggest that miR-107 may function as a tumor promoter in HCC.

## DISCUSSION

HCC is the most common type of primary liver cancer and is the third leading cause of cancer-related death [22]. Proliferation and metastasis, two of the most important hallmarks of cancer, are the leading lethal factors for malignant cancer, especially for HCC [1, 23, 24]. Recent studies have shown that miRNAs play a fundamental role in HCC, thereby opening a novel avenue for investigating the molecular mechanisms of HCC pathogenesis. Chang et al. [25] reported that overexpressed miR-331-3p promotes the proliferation and metastasis of HCC by mediating protein phosphatase. Lee et al. [26] suggested that miR-216b affects HCC growth and metastasis through the FGFR1/ERK signaling pathway. In the present study, our data showed that miR-107 acts as a tumor promoter in HCC by accelerating growth, both *in vitro* and *in vivo*, and exacerbating metastasis of human HCC cells. More importantly, CPEB3 was identified as a novel and functional target of miR-107, which acts as a tumor suppressor in HCC. Furthermore, we showed that miR-107 regulates the pathogenesis of HCC partially through the CPEB3/EGFR pathway. Therefore, our data present a novel mechanism for HCC progression.

To date, recent findings have shown that miR-107 was involved in various pathological processes including carcinogenesis. Song et al. [27] reported that miR-107 contributes to accelerating the proliferation of gastric cancer cells. While Zhou et al. [28] suggested that the miR-107 acts as a tumor-suppressor in cervical cancer. However, little is known about the role and underlying molecular mechanisms of miR-107 in human HCC. In this report, we explored the functional role of miR-107 in human HCC progression. A recent published study reported that miR-107 exhibits weak to moderate tumor suppressor potential in c-Myc and AKT/Ras mice [29]. These conclusions are not clear because the role of miR-107 in tumorigenesis is complicated and seems to be tissue type-dependent [27, 28], and the molecular mechanisms involved in the two studies may be different. To confirm the tumor promoter role of miR-107 in our study, the Huh7 and HepG2 human HCC cell lines were used to perform the MTT, transwell, colony formation and wound healing experiments (Figure 1). The *in vivo* experiments were also performed in the xenograft model (Figure 2). The transfection efficiency of all of the cells used in these tests were verified in advance (Figure 1A, 1D–2A). We demonstrated that overexpressing miR-107 contributes to proliferation both *in vitro* and *in vivo* in human HCC (Figure 1–2). Furthermore, the migration and invasion of human HCC cells were promoted (Figure 3). We tested the migration and invasion of both cell lines and obtained consistent results (data not shown).

At the molecular level, we performed luciferase assays and western blotting analyses to determine that miR-107 suppresses the expression of CPEB3 by targeting its 3′-UTR. CPEBs is a type of protein that binds to the specific element of the mRNA 3′-UTR to modulate cytoplasmic polyadenylation and acts as either a translational repressor or activator to regulate meiotic cell cycles [30–32]. The studies of CPEBs in disease are relatively rare, especially in cancer. Novoa et al. reported that CPEB1 and CPEB4 participates the progression of mitosis and regulated the cell cycles [32]. Bava et al. demonstrated that CPEB1 is involved in alternative 3′UTR formation and hence most likely modulates tumor progression [33]. Furthermore, Ortiz-Zapater et al. indicated that CPEB4 promotes tumor growth and vascularization in gliomas, which was the first study that directly linked CPEB4 to carcinogenesis [34]. To date, CPEB3 has been well established as playing critical roles in neuronal biological function, especially in memory activation [19, 20, 35]. Little is known about the functional role of CPEB3 beyond the nervous system [18]. Our findings showed that decreased CPEB3 expression significantly promotes the progression of HCC. This result was supported by a meta-analysis of microarray studies that showed dramatically decreased CPEB3 expression in HCC tissues versus healthy controls [18].

The last new finding of this study is that miR-107 may facilitate HCC pathogenesis through the CPEB3/EGFR axis. To further understand the molecular mechanism of miR-107 HCC promoter potential, western blotting was performed to test the potential signaling at the protein level. Because EGFR signaling has been demonstrated to be negatively regulated by CPEB3 in neurons [19], we suspected that decreased CPEB3 expression would enhance the EGFR level in HCC. Interestingly, we observed that the miR-107-mediated downregulation of the CPEB3 level increased the expression of EGFR, which is a regulating pivot in HCC [36]. In addition, the downstream signaling proteins AKT/pAKT and p21 were modulated as expected to facilitate the pathogenesis. We also tested the expression of PTEN, but found no significant difference that was dependent on the regulation of CPEB3 (data not shown). In conclusion, the newly identified miR-107/CPEB3 axis provides new insight into the pathogenesis of HCC and represents a novel, potential therapeutic target for the treatment of HCC. In addition, we performed the miR-107 ISH in clinical samples. The results support that miR-107 promotes tumor effects in HCC.

## MATERIALS AND METHODS

### Ethics statement

The investigations complied with ethical standards and the Declaration of Helsinki; they were performed according to national and international guidelines and were approved by the authors' institutional review board.

### Cell lines and culture conditions

Two human hepatocellular cancer cell lines (HepG2 and Huh7) and HEK293T cells were grown in DMEM (GIBCO Laboratories, Grand Island, NY, USA). The medium contained 10% fetal bovine serum, 100 U/ml penicillin G and 100 μg/ml streptomycin (GIBCO Laboratories, Grand Island, NY, USA). All cells were cultured at 37°C in a humidified incubator containing 5% CO_2_.

### Plasmid construction, oligonucleotide synthesis and transfection

The hsa-miR-107 mimic, miR-107 inhibitor, mimic negative control (NC mimic), inhibitor negative control (NC inhibitor) sequences and human CPEB3 siRNA were obtained from the Gene Pharma Company (Shanghai, China). The cells were transfected by Lipofectamine 2000 Transfection Reagent (Invitrogen, Carlsbad, CA, USA). For all related DNA sequences, see the Table 1.

**Table 1:** Primers for plasmid construction, qRT-PCR, and oligonucleotides

Primer function/target and name	Direction*	Sequence
Plasmid construction		
CPEB3 3′UTR WT	F	CTGAGCTCACTCGTGAGTAGGTGGCAGA
	R	GTTCTAGACATGCCTTCCTCCGGTCAAT
qPCR		
miR-107 stem-loop	RT	GTCGTATCCAGTGCGTGTCGTGGAGTCGGCAAT
		TGCACTGGATACGACTGATAG
U6	RT	CGCTTCACGAATTTGCGTGTCAT
miR-107	F	AGCAGCATTGTACAGGGCTATCA
	R	ATTGCGTGTCGTGGAGTCG
CPEB3	F	GAAAGGTAAACACTACCCTCCCA
	R	CCAGGAAGGCATTGTTAAGTGC
β-Actin	F	TACCTCATGAAGATCCTCACC
	R	TTTCGTGGATGCCACAGGAC
U6	F	GCTTCGGCAGCACATATACTAAAAT
	R	CGCTTCACGAATTTGCGTGTCAT
Oligonucleotides		
miR-107 mimic		AGCAGCAUUGUACAGGGCUAUCA
		AUAGCCCUGUACAAUGCUGCUUU
Negative Control (NC mimic)		UUCUCCGAACGUGUCACGUTT
		ACGUGACACGUUCGGAGAATT
miR-107 inhibitor		UGAUAGCCCUGUACAAUGCUGCU
Inhibitor NC		CAGUACUUUUGUGUAGUACAA
siCPEB3		GGACCGAUAAUGGUAACAATT
		UUGUUACCAUUAUCGGUCCTT
Negative Control (siNC)		UUCUCCGAACGUGUCACGUTT
		ACGUGACACGUUCGGAGAATT

TABLE Primers for plasmid construction, qRT-PCR, and oligonucleotides

* F, forward; R, reverse.

### Quantitative real-time PCR analysis (qRT-PCR)

Total RNA was extracted with TRIzol reagents (Invitrogen, Carlsbad, CA) according to the manufacturer's instructions. Subsequently, 1 μg of RNA was reverse transcribed into complementary DNA (cDNA) with random primers or a miR-107-specific stem-loop primer for CPEB3 and miR-107, respectively. With specific primers, the qRT-PCR was performed using an ABI 7500 PCR System (Applied Biosystems, Mannheim, Germany). The annealing temperature for CPEB3 and miR-107 was 60°C. Actin was used as the endogenous control for the detection of mRNA expression level, whereas U6 was used as the endogenous control for miRNA expression analysis. Relative quantification analysis was performed using the comparative CT (2^-ΔΔCT) method.

### Cell viability assay

Cell viability was assessed with the 3-(4, 5-dimethylthiazol-2-yl)-2, 5-diphenyltetrazolium bromide (MTT) assay. HepG2 or Huh7 cells were seeded in 96-well culture plates at a density of 5000 or 10000 per well. When the cells were 70–80% confluence, they were transfected with the oligonucleotide as required. After 1–4 days, the cells were stained with 20 μl of MTT (5 mg/ml in PBS)(Sigma, St Louis, MO, USA) for 4 h at 37°C. The cell medium was carefully aspirated and 150 μl of dimethyl sulfoxide (DMSO) was added to each well. Then, the cell plates were mildly shaked, and the absorbance was measured at 490 nm using a microtiter plate reader (TECAN, Männedorf, Switzerland).

### Colony formation assay

To investigate colony formation ability, HepG2 or Huh7 cells were transfected with miR-107 mimic, miR-107 inhibitor, or NC and subsequently seeded in 6-cm plates (1 000 cells/dish) and incubated for 2 weeks to allow for colony formation. The colonies were then fixed in methanol, stained with 0.1% crystal violet (Sigma, St Louis, MO, USA) and counted.

### Migration and invasion assay

To examine the migratory ability of cells *in vitro*, the transwell chamber assay was performed. Cells were placed in the upper chamber of a 24-well transwell unit (5 × 10^4^ cells/well) with 8-μm polycarbonate nucleopore filters (Corning Costar, Cambridge, MA). The upper compartment contained serum-free medium while the lower compartment contained medium with 10% fetal bovine serum; the cells were incubated for 24 h in a humidified atmosphere of 5% CO_2_ at 37°C. The cells adhering to the lower surface of the filter were fixed and counted. The cells from at least five representative fields were analyzed. For the invasion assay, the membrane of the transwell unit was coated with 40 μl Matrigel (BD Biosciences, San Jose, CA, USA) at 37°C for 4 h to form a reconstructed basement membrane. The cells were treated in the same way as the migration assay.

A wound healing assay was also applied to evaluate the cell migration ability. Cells were seeded in 3.5-cm plates and grown to a density of 70–80%. Then, a 200-μl pipette tip as used to create an artificial wound of scratched cells. The migrating distance was measured after 48 h.

### Western blotting analysis

Total cell or tissue extracts were extracted using cell lysis buffer followed by immunoblotting with anti-CPEB3 (1:500, Abcam, Cambridge, MA, USA), anti-AKT (1:1000, Cell Signaling Technology, Danvers, MA, USA), anti-p-AKT (1:2000, Cell Signaling Technology, Danvers, MA, USA), anti-EGFR (1:1000, Cell Signaling Technology, Danvers, MA, USA), anti-p-21 (1:1000, Cell Signaling Technology, Danvers, MA, USA), and anti-β-actin (1:4000, Santa Cruz Biotechnology, Santa Cruz, CA) as described previously [37].

### Luciferase activity assay

To construct a pmir-CPEB3–3′UTR plasmid containing the potential miR-107 binding sites, a 597-bp sequence was amplified and inserted into the SacI and XbaI sites of the pmir-GLO Dual Luciferase vector (Promega, Madison, WI, USA). This sequence contained the two predicted binding sites at 2973nt-2981nt and 3183nt-3190nt. The plasmid with mutant-type (MUT1, the first binding site is mutant; MUT2, the second binding site is mutant and MUT3, both two binding sites are mutant) were inserted downstream of the firefly the pmirGLO Dual-Luciferase vector. HEK293 cells were used to measure luciferase activity. When grown to 60–70% confluence, the cells were co-transfected with a 100-ng Luciferase plasmid along with a 650-ng miR-107 mimic or a NC mimic as described above. After incubation for 24 h at 37°C, the luciferase activity was determined using the Dual Luciferase Reporter 1 000 Assay System (Promega, Madison, WI, USA).

### Mouse experiments

Male athymic BALB/c nu/nu mice (aged 4 weeks) were purchased from Shanghai Laboratory Animal Center (Shanghai, China). All animal procedures were in accordance with Harbin Medical University Institutional Animal Care and Use Committee guidelines. The animals were housed as described previously [37]. Then, 5 × 10^6^ HepG2 cells that were transfected with miR-107 or CPEB3 siRNA were injected subcutaneously into the right flank of the nude mice. Tumor size was measured using a caliper every 3 days. Both the maximum (L) and minimum (W) lengths of the tumors were measured, and the tumor size was calculated as ½LW2. After 21 days, the mice were sacrificed and photographed. Tumors were harvested and weighed, and five animals were included in each group.

### Tissue microarray assay

The HCC Tissue Microarray (TMA) experiments were performed by Shanghai Outdo Biotech Co., Ltd (Cat. No. Hliv-HCC060PG-01). For the TMAs, there were a total of 30 HCC cancer patient cases, paired as tumor-bearing tissues and adjacent tumor-free tissues from the same patient. All tissues were re-examined using a microscope by an experienced pathologist after the tissues were transferred from a local hospital; the TNM stage was determined in each patient.

ISH of miR-107 with microarrays was performed by Shanghai Outdo Biotech Co., Ltd. The hsa-miR-107 miRCURY LNA Detection probe, 5′-DIG and 3′-DIG labeled, was used as the microRNA probe (Exiqon, 18015–15).

### Image analysis

The positivity plots in TMA were measured by analyzing the staining signal intensity using Aperio image scope v11 (Aperio, USA). The brown staining (positive) in the intensely stained image and the blue staining (negative) in the least intensely stained area were selected for further data processing. The subsequent staining intensity was measured as the densitometry of the digital image (× 200), and the pixels that were counted positive were transformed into three intensity bins.

The application “Positive Pixel Count V9” of Aperio image scope v11 was used to select the areas of interest from each image, and the image data were then translated into numerical data, such as the positive signal intensity, the negative signal intensity and the number of positive and negative signals. The average intensity, which is the ratio of the sum of the positive signal intensities (weak positive, positive and strong positive) to the sum of the number of positive signals (weak positive, positive and strong positive), was calculated and used for further statistical analysis.

### Statistical analysis

SPSS V20.0 was used for the statistical analysis. All values are expressed as the mean ± SEM, and all experiments were repeated at least three times. Student *t*-tests were used to determine the statistical significance of the differences between groups. Comparative *t*-test was used for the clinical sample analysis. Differences with *p* < 0.05 were considered significant (*p < 0.05, **p < 0.01, ***p < 0.001).
